# Flotillin-1 Interacts With and Sustains the Surface Levels of TRPV2 Channel

**DOI:** 10.3389/fcell.2021.634160

**Published:** 2021-02-09

**Authors:** Juan Hu, Yue Gao, Qian Huang, Yuanyuan Wang, Xiaoyi Mo, Peiyu Wang, Youjing Zhang, Chang Xie, Dongdong Li, Jing Yao

**Affiliations:** ^1^State Key Laboratory of Virology, Hubei Key Laboratory of Cell Homeostasis, College of Life Sciences, Frontier Science Center for Immunology and Metabolism, Wuhan University, Wuhan, China; ^2^Institute of Biology Paris Seine, Neuroscience Paris Seine, CNRS UMR8246, INSERM U1130, Sorbonne Université, Paris, France

**Keywords:** TRPV2, flotillin-1, protein–protein interactions, protein stability, thermal sensation

## Abstract

Transient receptor potential vanilloid subtype 2 (TRPV2) channel is a polymodal receptor regulating neuronal development, cardiac function, immunity and oncogenesis. The activity of TRPV2 is regulated by the molecular interactions in the subplasmalemmel signaling complex. Here by yeast two-hybrid screening of a cDNA library of mouse dorsal root ganglia (DRG) and patch clamp electrophysiology, we identified that flotillin-1, the lipid raft-associated protein, interacts with TRPV2 channel and regulates its function. The interaction between TRPV2 and flotillin-1 was validated through co-immuoprecipitation *in situ* using endogenous DRG neurons and the recombinant expression model in HEK 293T cells. Fluorescent imaging and bimolecular fluorescence complementation (BiFC) further revealed that flotillin-1 and TRPV2 formed a functional complex on the cell membrane. The presence of flotillin-1 enhanced the whole-cell current density of TRPV2 via increasing its surface expression levels. Using site-specific mapping, we also uncovered that the SPFH (stomatin, prohibitin, flotillin, and HflK/C) domain of flotillin-1 interacted with TRPV2 N-termini and transmembrane domains 1–4, respectively. Our findings therefore demonstrate that flotillin-1 is a key element in TRPV2 signaling complex and modulates its cellular response.

## Introduction

Transient receptor potential vanilloid type 2 (TRPV2) was firstly identified in 1999. It has been proposed to mediate responses to noxious thermal (52°C) stimuli in medium- to large-diameter sensory neurons (Caterina et al., 1999). TRPV2 is a Ca^2+^-permeable and polymodal channel modulated by mechanical stretch, heat, osmotic swelling, and endogenous and exogenous chemical modulators such as 2-aminoethoxydiphenyl borate (2-APB), cannabinoids, probenecid, amiloride and SKF96365 (Bang et al., 2007; Colton and Zhu, 2007; Juvin et al., 2007; Qin et al., 2008). TRPV2 is a well-conserved channel protein expressed in almost all tissues, such as the central and peripheral nervous systems, the immune system, and the muscular system. Structurally, mammalian TRPV2 consists of a 6 transmembrane (TM)-domain structure flanked by a large cytosolic N- and C- terminus with a pore-forming loop between the fifth and sixth TM domains (Huynh et al., 2016). Recent research on TRPV2 has made remarkable progress, especially in terms of the discovery of its physio-pathological functions. In addition to being a thermosensor for noxious heat, the widespread distribution of TRPV2 has been implicated in developing axon outgrowth (Shibasaki et al., 2010), various osmo- or mechanosensation (Donate-Macian et al., 2019), physiological cardiac structure maintenance and function (Katanosaka et al., 2014), insulin secretion (Aoyagi et al., 2010), proinflammatory processes (Entin-Meer et al., 2017), immune activity (Link et al., 2010; Santoni et al., 2013).

TRPV2 channel is organized in signaling complexes, where its function is regulated by protein–protein interactions. For instance, TRPV2 channel biogenesis is associated with post-translational modification such as recombinase gene activator protein (RGA)-related glycosylation (Stokes et al., 2005) and protein kinase A (PKA)-mediated phosphorylation (Stokes et al., 2004). In addition, certain signaling molecules have been documented to regulate the channel expression. TRPV2 protein levels are upregulated by nerve growth factor (NGF) via the mitogen-activated protein kinase (MAPK) signaling pathway (Cohen et al., 2015). Antiaging gene Klotho enhances glucose-induced insulin secretion by increasing plasma membrane levels of TRPV2 and thus calcium influx (Lin and Sun, 2012). Other modulators have also been shown to affect TRPV2 function. For example, phosphatidylinositol 4,5-bisphosphate (PIP2) depletion contributes to Ca^2+^-dependent desensitization of TRPV2 (Mercado et al., 2010). Inflammatory factors like TNF−α facilitate TRPV2 responses in human dental pulp cells (Liu et al., 2019). Further identification of binding partners will help to deepen the understanding of the regulatory mechanisms of TRPV2.

The flotillin (FLOT)/reggie protein family is integral membrane element and contains two isoforms: flotillin-1 (reggie-2) and flotillin-2 (reggie-1) (Schulte et al., 1997; Browman et al., 2007). Their C-terminal flotillin domains are required for the oligomerization, while the N-terminal SPFH (stomatin, prohibitin, flotillin, and HflK/C) domain is mainly responsible for membrane interactions (Solis et al., 2007; Bodin et al., 2014). Flotillin-1 is highly expressed in the nervous system and has been identified as an evolutionarily conserved protein involved in learning and memory (Monje et al., 2013). Flotillin-1 participates in various cellular functions, including cell adhesion, actin cytoskeleton reorganization, endocytosis, phagocytosis and the transduction of cellular signals (Langhorst et al., 2005). Moreover, flotillins are considered to be scaffolding proteins of lipid rafts and are preferentially associated with many other proteins. Flotillins interact with Src family kinase Fyn and contribute to the formation of signal transduction centers in adipocytes, T lymphocytes, and neurons (Stuermer et al., 2001; Liu et al., 2005). Flotillin-1 interacts with CAP, Vinexin α and ArgBP2, respectively, and regulates the organization of the actin cytoskeleton (Kimura et al., 2001). Cornfine et al. (2011) reported that flotillin-2 interacted with kinesin KIF9 and regulated matrix degradation by macrophage podosomes. In addition, flotillins have also been suggested to be associated with several ion channels, including the NR2A and NR2B subunits of NMDA receptors (Swanwick et al., 2009), Nav1.5 channels (Kessler et al., 2019), Kv2.1 channels (Liu et al., 2016), voltage-gated calcium channels (Robinson et al., 2010), calcium-activated chloride channels (Sones et al., 2010), aquaporin 5 (Wang et al., 2011), GABAA receptor (Nothdurfter et al., 2013), and the purinergic P2X3 receptor (Vacca et al., 2004) in different tissues. Nevertheless, the role of flotillins in regulating TRPV2 functions remains unaddressed.

Here combining yeast two-hybrid screening, immuno-precipitation and BiFC imaging, we demonstrate that flotillin-1 interacts with TRPV2 channel at the protein level. Heterologous co-expression of flotillin-1 increased the whole-cell current density of TRPV2 as a result of enhanced surface expression. Moreover, we show that the N-terminus and TM domains (1–4) of TRPV2 interact with the SPFH domain of flotillin-1 in the multiprotein assembly. Our findings thus indicate that flotillin-1 regulates TRPV2 channel cellular response by sustaining its surface stability.

## Materials and Methods

### cDNA Constructs

The wild-type rat TRPV2 and TRPV1 cDNAs were kindly provided by Dr. Feng Qin (State University of New York at Buffalo, Buffalo). The pFlotillin-1-NsfGFP, pFlotillin-1-CsfGFP, pFlotillin-2-NsfGFP, pFlotillin-2-CsfGFP, pCsfGFP and pNsfGFP cDNAs were generously provided by Dr. Yu Ding (Fudan University, China). The full-length of flotillin-1 and flotillin-2 were cloned from the mouse dorsal root ganglion (DRG) cDNA library. For the expression in mammalian cells, the cDNAs of TRPV2, flotillin-1, and flotillin-2 were introduced into p3 × Flag-cmv-7.1, pEGFP-N1, or pcDNA5-HA, as indicated. The Flag-tags or HA-tags were fused to the N terminus of the target proteins, and EGFP was tagged to the C terminus of the proteins of interest. All recombinant constructs and mutations were carried out using the overlap-extension polymerase chain reaction (PCR) method. The resulting constructs and mutations were then verified by DNA sequencing. Oligo DNAs targeting flotillin-1 were synthesized, annealed and inserted into pLenti-GFP vector. The sequences of shRNA against flotillin-1 are as follows: #1, 5′- GGGACTATGAGCTGAAGAA-3′; #2, 5′-GCGTGGTTAGCTACACTTT-3′.

### Cell Culture and Transfection

All mice were housed in the specific pathogen-free animal facility at Wuhan University and all animal experiments were in accordance with protocols approved by the Institutional Animal Care and Use Committee of Wuhan University (NO. WDSKY0201804). Primary culture of DRG neurons was established following enzymatically and mechanically dissociation of the ganglia as described before with minor modification (Tian et al., 2019). In brief, 6 to 8-week-old wild-type C57 BL/6 mice were deeply anesthetized and then decapitated. L3–L4 DRGs together with dorsal-ventral roots and attached spinal nerves were taken out from the spinal column. After removing the attached nerves and surrounding connective tissues, DRG neurons were rinsed with ice-cold phosphate buffer saline (PBS). Then total RNA was extracted from the intact DRG for yeast cDNA library construction using TRIzol (Life Technologies) following the manufacturer’s instructions or directly homogenized DRG neurons for immunoprecipitation experiment.

HEK 293T and ND7/23 cells were cultured in Dulbecco’s Modified Eagle’s Medium (DMEM, Thermo Fisher Scientific, MA, United States) containing 4.5 mg/ml glucose, 10% heat-inactivated fetal bovine serum (FBS, Gibco, Thermo Fisher Scientific), 50 units/ml penicillin, and 50 μg/ml streptomycin, and were incubated at 37°C in a humidified incubator gassed with 5% CO_2_. Cells grown into ∼80% confluence were transfected with the desired DNA constructs using either the standard calcium phosphate precipitation method or Lipofectamine 2000 (Invitrogen) following the protocol provided by the manufacturer. Transfected HEK 293T cells were reseeded on 12 mm round glass coverslips coated by poly-L-lysine for electrophysiological experiments. Experiments took place usually 12–24 h after transfection.

### Generation of TRPV2 Stable Cell Lines

For generation of stable cell lines, HEK 293T cells were transiently transfected with pHAGE-6tag-puro-TRPV2 using Lipofectamine 2000 (Invitrogen). Transfected cells were grown for 2 weeks in puromycin in order to select for stable expressing cells. Non-transfected cells were killed by additions of puromycin (2 μg/ml) 2 days after treatment. The surviving cells were resistant to puromycin, and these cells were successfully expressed with *Trpv2* gene. Medium was replaced every 2–3 days until single colonies were formed, then the stable cell lines were placed in a complete medium containing 1 μg/ml puromycin.

### Yeast Two-Hybrid Assay

Yeast two-hybrid screen was conducted using the Matchmaker GAL4-based two-hybrid system (Clontech) as previously described (Wang et al., 2020). Briefly, the mouse DRG cDNA library was fused to the GAL4 activation domain of plasmid pGADT7 (Clontech). The titer of the primary cDNA library was calculated using the number of clones on plates. Colony PCR was used to verify the size of the inserted fragments in the library. Bait plasmid was constructed by introducing the N-terminal (aa 1–387) of TRPV2 into the GAL4 DNA binding domain of the pGBKT7 vector (Clontech). The pGBK-bait was transformed into the yeast strain Y187 and pGAD-preys into AH109. Cytotoxicity and self-activation activity were determined via observing yeast clone growth and size. Diploid yeast cells from yeast mating were selected on the triple dropout medium (TDO: SD/-His/-Leu/-Trp). Replica plate colonies were then transferred onto quadruple dropout medium (QDO: SD/-Ade/-His/-Leu/-Trp) containing *X*-α-Gal (4 mg/mL). X-α-gal was used as substrate for colorimetric detection of α-galactosidase activity. Plates were incubated at 30°C for 7 days. Plasmids extracted from the positive clones were transformed into *E. coli* cloning host DH5α to be amplified, and samples were then sequenced individually. BLAST comparisons and other bioinformatics methods were applied for sequence analysis. Thereafter, the bait and prey plasmids in different combinations were sequentially co-transformed into yeast stain AH109 and selected on double dropout medium (DDO: SD/-Leu/-Trp) and incubated at 30°C for 3–4 days. Then the positive clones were diluted and equally coated onto SD/-Leu/-Trp/-His/-Ade medium with *X*-α-gal and SD/-Leu/-Trp medium and cultured at 30°C for 3–4 days. In parallel, the combination of pGBKT7-53/pGADT7-T and pGBKT7-Lam/pGADT7-T were used as positive and negative controls, respectively.

### Chemicals and Antibodies

Key antibodies and reagents used in this study were as bellow.

**Table:** 

**Antibodies or Reagents**	**Source**	**Identifier**
Rabbit anti-TRPV2	Alomone labs	Cat#ACC-032
Rabbit anti-Flotillin-1	Thermo scientific	Cat#PA5-28118
Rabbit anti-Na^+^/K^+^ ATPase	Abcam	Cat#ab76020
Mouse anti-Flag	MBL	Cat#M185-3L
Rabbit anti-Flag	Proteintech	Cat#20543-1-AP
Mouse anti-GFP	Tianjin sungene biotech	Cat#KM8009
Rabbit anti-GFP	Solarbio life science	Cat#B1025F
Mouse anti-HA	MBL	Cat#M180-3
Rabbit anti-HA	Earthox	Cat#E02218002
Anti-Flag Affinity Gel	Bimake	Cat#B23100
Anti-HA Affinity Gel	Bimake	Cat#B23301
Goat anti-mouse IgG (H + L)	Jackson immunoresearch	Cat#115-035-003
Goat anti-rabbit IgG (H + L)	Jackson immunoresearch	Cat#111-005-003
EZ-Link Sulfo-NHS-LC-Biotin	Thermo fisher scientific	Cat#21335
High Capacity NeutrAvidin^TM^ Agarose	Thermo fisher scientific	Cat#29202
Cycloheximide	Merck	Cat#239763-M
Puromycin	Merck	Cat#P8833

### Immunoprecipitation and Western Blot

Immunoprecipitation were performed as previously described (Wang et al., 2020). In brief, cells were harvested 24 h post-transfection and lysed in Non-idet P-40 lysis buffer containing 150 mM NaCl, 1 mM EDTA, 20 mM Tris–HCl, 1% Non-idet P-40, and 1% complete protease and phosphatase inhibitor cocktail (Bimake). Lysates were cleared of debris by centrifugation at 14,000 rpm for 10 min. The supernatants were collected and immunoprecipitated with the indicated antibodies and Protein G agarose beads for 2 h. The immunoprecipitants were washed with 1 ml lysis buffer containing 500 mM NaCl for three times followed by immunoblotting assay. Samples were loaded into 4–20% or 12% SDS-PAGE gels and electrophoretically separated according to the manufacturer’s instructions (125 V for 60 min in Running Buffer).

### Fluorescence Confocal Microscopy

HEK 293T cells transfected with the desired plasmids were fixed with 4% paraformaldehyde (Biosharp, BL539A) for 10 min at 4°C and the nuclei were counterstained with DAPI (Merck, D5492) for 15 min at room temperature in the dark, and washed three times with phosphate buffered saline (PBS). The cells were scanned and images were collected using a Leica SP8 confocal microscopy (63× oil objective NA 1.35).

### Surface Biotinylation Assay

Surface biotinylation was performed following established protocols (Wang et al., 2018). Cells were firstly washed three times with the ice-cold PBS solution supplemented with 1 mM MgCl_2_ and 2.5 mM CaCl_2_ (pH 8.0). Then Sulfo-NHS-LC-Biotin (0.25 mg/ml; Thermo Scientific, Waltham, MA, United States) was added into the same solution and incubated with cells at 4°C for 30 min with gentle rocking. The unbound biotin group was quenched by the addition of 0.1 M glycine for 20 min at 4°C. Biotin-labeled proteins were isolated by incubating whole cell lysates with NeutrAvidin agarose beads (Thermo Fisher Scientific) overnight at 4°C with rocking. The beads were washed three times with PBS (pH 8.0) and bound proteins were eluted with the boiling SDS sample buffer and used for immunoblotting.

### Electrophysiology

Conventional whole-cell patch-clamp recording methods was used. For the recombinant expressing system, green fluorescent from EGFP was used as a marker for gene expression. Currents were amplified using an Axopatch 200B amplifier (Molecular Devices, Sunnyvale, CA, United States) and recorded through a BNC-2090/MIO acquisition system (National Instruments, Austin, TX, United States) using QStudio developed by Dr. Feng Qin at State University of New York at Buffalo. Whole-cell recordings were typically sampled at 5 kHz and low-pass filtered at 1 kHz.

Bath solution for whole-cell recording consisted of (mM): 140 NaCl, 5 KCl, 3 EGTA, and 10 HEPES, pH 7.4 adjusted with NaOH. Electrodes were filled with (mM): 150 CsCl, 5 EGTA, 10 HEPES, pH 7.4 adjusted with CsOH. Recording pipettes were made from borosilicate glass capillaries (World Precision Instruments), and fire-polished to a resistance between 2 and 4 MΩ when filled with electrode saline. 2-Aminoethyl diphenylborinate (2-APB) was dissolved in DMSO to make a stock solution. All the stocks were diluted in the bath solution to the desired concentrations right before the experiment. Exchange of external solutions was performed using a gravity-driven local perfusion system. As determined by the conductance tests, the solution around a patch under study was fully controlled by the application of a solution with a flow rate of 100 μl/min or greater. All pharmacological experiments met this criterion. Unless otherwise noted, all chemicals were purchased from Sigma (Millipore Sigma, St. Louis, MO, United States). All patch-clamp recordings were made at room temperature (22–24°C) except for temperature stimulation.

### Ultrafast Temperature Jump Achievement

A single emitter infrared laser diode (1,550 nm) was designed to produce temperature jumps as previously described (Yao et al., 2009). The laser diode was driven by a pulsed quasi-CW current power supply (Stone Laser, Beijing, China), and pulsing of the controller was controlled from a computer through BNC-2090/MIO data acquisition card, which was also responsible for patch-clamp recordings. The launched laser beam was transmitted by a multimode fiber with a core diameter of 100 μm. A blue laser line (460 nm) was coupled into the same fiber to aid alignment. Temperature was calibrated offline from the electrode current based on the temperature dependence of electrolyte conductivity. The temperature threshold for heat activation of TRPV2 was determined as the temperature at which the slow inward current was elicited.

### Data Analysis

Densitometry was performed using ImageJ software for the quantitative analysis of the bands on the western blots. Electrophysiological data were analyzed offline with Qstudio developed by Dr. Feng Qin at State University of New York at Buffalo, Clampfit (Molecular Devices, Sunnyvale, CA, United States), IGOR (Wavemetrics, Lake Oswego, OR, United States), SigmaPlot (SPSS Science, Chicago, IL, United States) and OriginPro (OriginLab Corporation, MA, United States). For concentration response analysis, the modified Hill equation was used: *Y* = A1 + (A2 − A1)/[1 + 10 ^ (logEC_50_ − *X*) ^∗^ *n*_*H*_], in which EC_50_ is the half maximal effective concentration, and *n*_*H*_ is the Hill coefficient. Unless stated otherwise, the summary data are presented as mean ± standard error (s.e.m.) or mean ± standard deviation (S.D.) as indicated, with statistical significance assessed using unpaired student *t*-test for two-group comparison. Significant difference is indicated by a *p*-value less than 0.05 (^∗^*p* < 0.05, ^∗∗^*p* < 0.01, ^∗∗∗^*p* < 0.001).

## Results

### Flotillin-1 Physically Interacts With TRPV2 Channel

**FIGURE 1:**
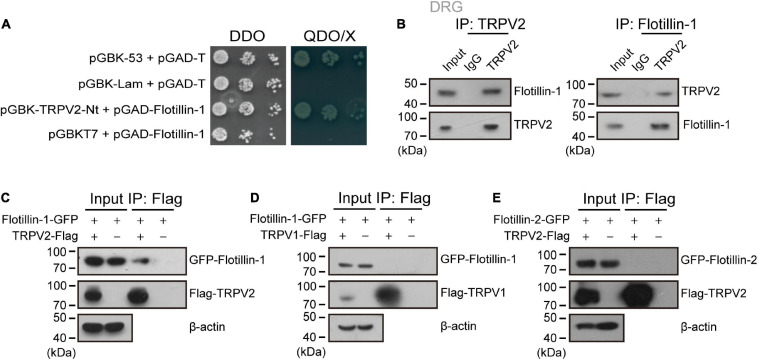
Flotillin-1 physically interacts with TRPV2. **(A)** Yeast two-hybrid results showing the interaction between TRPV2 N-terminus (V2-Nt) and flotillin-1. The bait and prey constructs were co-transformed into yeast strain AH109, and the transformed yeast diploids were grown on DDO for 2 days and QDO for 5 days, respectively. The 53-T and Lam-T were served as positive and negative controls, respectively. DDO, SD-Leu/-Trp medium; QDO, SD-Leu/-Trp/-His/-Ade medium; X, *X*-α-gal. **(B)** Immunoprecipitation (with anti-TRPV2, anti-Flotillin-1, or IgG as a control) and immunoblot analysis (with anti-TRPV2 or anti-Flotillin-1) of the mouse DRG neurons. **(C)** Interaction between flotillin-1 and TRPV2. HEK 293T cells were transfected with plasmids as indicated. Immunoprecipitation (IP, with anti-Flag) and immunoblot analysis (with anti-GFP and anti-Flag) of the interaction between TRPV2 and flotillin-1. **(D)** No interaction between flotillin-1 and TRPV1. Immunoprecipitation (with anti-Flag) and immunoblot analysis (with anti-GFP and anti-Flag) of HEK 293T cells transfected with plasmids encoding Flag-tagged TRPV1 and GFP-tagged flotillin-1. **(E)** No interaction between TRPV2 and flotillin-2. Immunoprecipitation (with anti-Flag) and immunoblot analysis (with anti-GFP and anti-Flag) of HEK 293T cells transfected with plasmids encoding Flag-tagged TRPV2 and GFP-tagged flotillin-2. Data are representative of three independent experiments at least.

In an attempt to identify biomolecular partners directly interacting with TRPV2 channel, we embarked on a yeast two-hybrid approach. Specifically, we screened a cDNA library prepared from mouse DRG using the cytosolic N-terminus of mouse TRPV2 as a bait. In the screen, 36 positive clones were subjected to sequencing. One of the positive clones harbored a 747-bp cDNA fragment that corresponds to amino acid residues 1–249 of flotillin-1. We thus chose flotillin-1 as the target protein. To validate the interaction, flotillin-1-pGADT7 was co-transformed with TRPV2-Nt-pGBKT7 into the yeast reporter strain AH109, and the transformants at serial dilutions were cultured under a SD/-Ade/-His/-Leu/-Trp selective condition and subjected to *X*-α-gal assay. In addition, the 53-T and Lam-T were also expressed and served as positive and negative controls, respectively. As illustrated in Figure 1A, only cells containing both plasmids encoding flotillin-1 and TRPV2-Nt or the combination of pGBK-53 and pGAD-T, but not the control cells, were able to grow in the selective medium. This result indicates that flotillin-1 interacts with the N-terminus of TRPV2.

We next determined whether flotillin-1 interacted with TRPV2 in endogenous adult DRG neurons using coimmunoprecipitation (co-IP) strategy. As shown in Figure 1B, in primary cultures of mouse DRG neurons, flotillin-1 robustly coimmunoprecipitated with TRPV2 and vice versa. Further, in mammalian HEK 293T cells, flotillin-1-GFP was also co-immunoprecipitated by TRPV2-Flag with Flag beads, confirming that flotillin-1 associated with the full length of TRPV2 (Figure 1C). However, TRPV1-Flag could not be precipitated by flotillin-1-GFP with Flag beads, implying that flotillin-1 does not interact with TRPV1 (Figure 1D).

Flotillin-1 and flotillin-2 usually form a heterodimer or oligomer complex (Liu et al., 2016; Gauthier-Rouvière et al., 2020). Thus, it is interesting to investigate whether flotillin-2 also interacted with TRPV2 channel. To this aim, co-immunoprecipitation was performed in HEK 293T cells with ectopically expressed flotillin-2 and full-length of TRPV2. As shown in Figure 1E, there was no interaction between flotillin-2 and TRPV2. Therefore, these observations imply that flotillin-1 physically interacts with TRPV2 channel.

### Visualization of Flotillin-1-TRPV2 Complex by Immunofluorescent Imaging and BiFC

**FIGURE 2:**
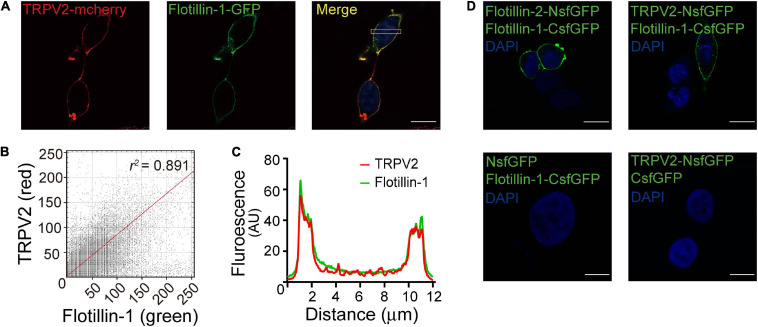
Fluorescence images show co-localization of TRPV2 and flotillin-1. **(A)** Co-localization of TRPV2 and flotillin-1. HEK 293T cells were co-transfected with mcherry-TRPV2 (red) and GFP-flotillin-1 (green) and examined with confocal microscopy. In the merged images, the extensive co-localization of the two proteins was represented by yellow. **(B)** The fluorescence intensity scatterplot showed a clear linear relationship, with a Pearson’s correlation coefficient value of 0.891. **(C)** The plot of fluorescence intensities between the red and green pixels along a section through the cell body as indicated in **(A)** indicated a well-correlated relationship between green and red. **(D)** Validation of the interaction between TRPV2 and flotillin-1 by bimolecular fluorescence complementation (BiFC) analysis. The confocal images of HEK 293T cells co-expressed with the indicated constructs, showing that strong fluorescence at the cell membrane region for that co-expressing TRPV2-NsfGFP and flotillin-1-CsfGFP, which is line with the green fluorescence observed from the BiFC complex formed by flotillin-1-CsfGFP and flotillin-2-NsfGFP. No detectable sfGFP fluorescence for HEK 293T cells co-expressed with NsfGFP and flotillin-1-CsfGFP or the combination of TRPV2-NsfGFP and CsfGFP. Nuclei were stained with DAPI. Scale bar: 10 μm. Data are representative of three independent experiments.

To further confirm the interaction between flotillin-1 and TRPV2, we co-expressed red fluorescent TRPV2-mcherry and green fluorescent flotillin-1-GFP in HEK 293T cells. Fluorescent imaging by confocal microscope showed apparent co-localization between TRPV2 and flotillin-1 (Figure 2A), as confirmed by quantitative analysis (Pearson’s coefficient = 0.891, Figures 2B,C).

Next, we employed the high-throughput bimolecular fluorescence complementation (BiFC) technique that enables the direct visualization of molecular interactions in the cell. Briefly, GFP molecule is splitted into two fragments that individually lack fluorescence and then conjugated to different proteins. Only when the two conjugated proteins interact at the molecular level, GFP fluorescence could be reconstituted. Considering that flotillin-1 and flotillin-2 have been demonstrated to form both homo- and hetero-oligomeric complexes (Babuke et al., 2009), a pair of BiFC constructs (flotillin-2-NsfGFP and flotillin-1-CsfGFP) were co-transfected into HEK 293 cells as a positive control. As expected, this produced GFP green fluorescence on the cell membrane (Figure 2D). In contrast, in cells expressing the negative control constructs (flotillin-1-CsfGFP vs. NsfGFP or TRPV2-NsfGFP vs. CsfGFP), we observed no fluorescent signals (Figure 2D). Next, the similar approach was carried out to evaluate the interaction between flotillin-1 and TRPV2. Co-expression of TRPV2-NsfGFP and flotillin-1-CsfGFP in HEK 293T cells displayed significant green fluorescence on cell surface (Figure 2D), thus validating the molecular combination between flotillin-1 and TRPV2.

### Flotillin-1 Increases Current Density of TRPV2

**FIGURE 3:**
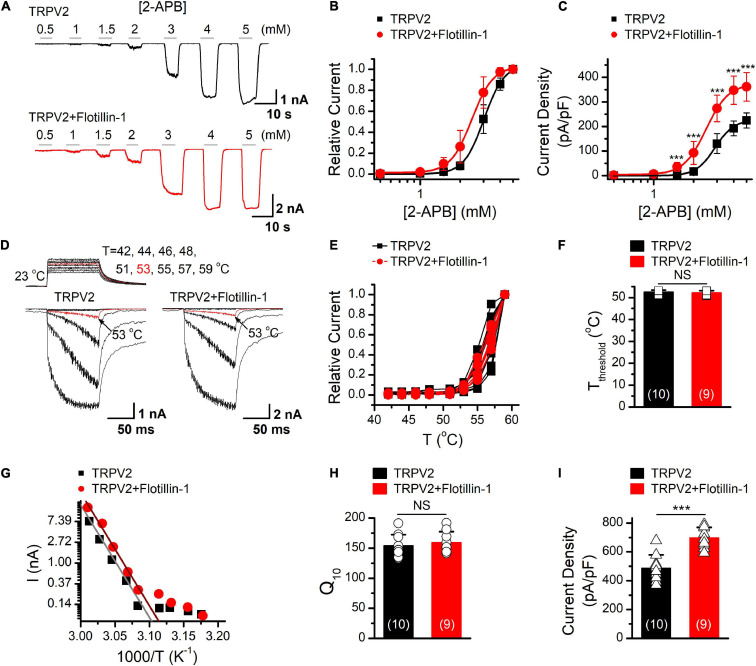
Flotillin-1 enhances TRPV2 activity. **(A)** Representative whole-cell recording in HEK 293T cells expressed TRPV2 alone (*upper*), and TRPV2 + flotillin-1 (*lower*), showing that 2-APB activated TRPV2 channels in a dose-dependent manner. Holding potential was –60 mV. **(B)** Dose–response curves for 2-APB-evoked currents. Solid lines indicate fits with the Hill equation, which yielded EC_50_ = 3.01 ± 0.04 mM, *n*_*H*_ = 5.72 ± 0.4 for TRPV2 (*n* = 8); and EC_50_ = 2.44 ± 0.04 mM, *n*_*H*_ = 5.43 ± 0.32 for TRPV2 + flotillin-1 (*n* = 8). **(C)** Summary plot of current density. The current densities evoked by 2-APB were determined by normalizing the membrane peak current by membrane capacitance (*n* = 8). ***P < 0.001. **(D)** Representative responses to a family of temperature jumps ranging from 42 to 57°C for HEK 293T cells that expressed TRPV2 alone (left), and TRPV2 + flotillin-1 (right). Temperature pulses stepped from room temperature were generated by laser irradiation were 100 ms long and had a rise time of 2 ms. Temperatures were calibrated offline from the pipette current using the temperature dependence of electrolyte conductivity. The red traces indicate the response at 53°C. **(E)** Temperature response curves for TRPV2 (black), TRPV2 + flotillin-1 (red), measured from the maximal currents at the end of temperature steps. Each curve represents measurements from an individual cell and the responses were normalized to the maximum responses at 59°C. **(F)** Comparison of temperature threshold for heat activation of TRPV2. Different symbols represent individual data points. The mean temperature thresholds (*T*_*threshold*_) of activation were 52.5 ± 0.8°C for TRPV2 (*n* = 10), 52.1 ± 0.9°C for TRPV2 + flotillin-1 (*n* = 9). *P* = 0.335. **(G)** Arrhenius plot of steady-state currents shown in **(D)**. The major component of the reflection that represents the strong temperature dependence was fitted to a linear equation. **(H)** Comparison of temperature dependence of TRPV2 and TRPV2 + flotillin-1. The values of Q10 derived from the linear fits in Arrhenius plot were as following, Q10 = 154.1 ± 18.2 for TRPV2 (*n* = 10), Q10 = 159.1 ± 18.2 for TRPV2 + flotillin-1 (*n* = 9). Colored symbols indicate individual data points. *P* = 0.566. **(I)** Summary of current density for temperature (59°C)-activated TRPV2 currents at –60 mV are shown. The current densities were 487.9 ± 90.8 pA/pF for TRPV2 (*n* = 10) and 697.9 ± 71.5 pA/pF for TRPV2 + flotillin-1 (*n* = 9); ***p < 0.001. Error bars represent SD.

As having confirmed that flotillin-1 associates with TRPV2, we next evaluated the functional consequence by patch clamp electrophysiology. Figure 3A shows representative traces of whole-cell currents recorded from HEK 293T cells expressing TRPV2 alone or TRPV2 and flotillin-1. With the cells held at −60 mV, 2-APB-evoked whole-cell currents were recorded in response to different doses of 2-APB. The concentration-response curves to 2-APB were fitted with a Hill equation, and the corresponding EC_50_ values and Hill coefficients (*n*_*H*_) for TRPV2 and TRPV2 co-expressed with flotillin-1 were not significantly changed by the presence of flotillin-1 (Figure 3B, EC_50_ = 3.01 ± 0.04 mM, *n*_*H*_ = 5.72 ± 0.4 for TRPV2 and EC_50_ = 2.44 ± 0.04 mM, *n*_*H*_ = 5.43 ± 0.32 for TRPV2 + flotillin-1). However, the TRPV2 current density calculated from steady-state currents yielded a remarkable increase in the presence of flotillin-1 as compared with recordings without flotillin-1, e.g., 361.4 ± 57.9 pA/pF (*n* = 8) versus 224.6 ± 30.7 pA/pF (*n* = 8) evoked by 5 mM 2-APB (Figure 3C).

TRPV2 channel is also a thermal sensor responding to noxious temperature for pain initiation. We next explored whether flotllin-1 modulates TRPV2 thermal responses. We used an infrared laser diode as a heat source to increase single cell temperatures under millisecond. HEK 293T cells expressing TRPV2 were held at −60 mV when the temperature jumps were delivered (Figure 3D). Plotting the relative responses against the step temperatures, we observed that the thermal activation profiles remained same for TRPV2 channels expressed with or without Flotillin-1 (Figures 3E,F). Using an Arrhenius plot of the amplitudes (Figure 3G), we determined the temperature coefficient (Q_10_) for TRPV2 (Figure 3H) and found no change in the presence of flotillin-1 (Figure 3F). By calculating the current density, however, we observed that flotillin-1 co-expression led to a significant increase in TRPV2 current density evoked by temperature jumps (Figure 3I). Together, our data suggests that flotillin-1 upregulates the current density of TRPV2 without altering the sensitivity to agonistic or thermal activations.

### Flotillin-1 Increases Membrane Expression of TRPV2

**FIGURE 4:**
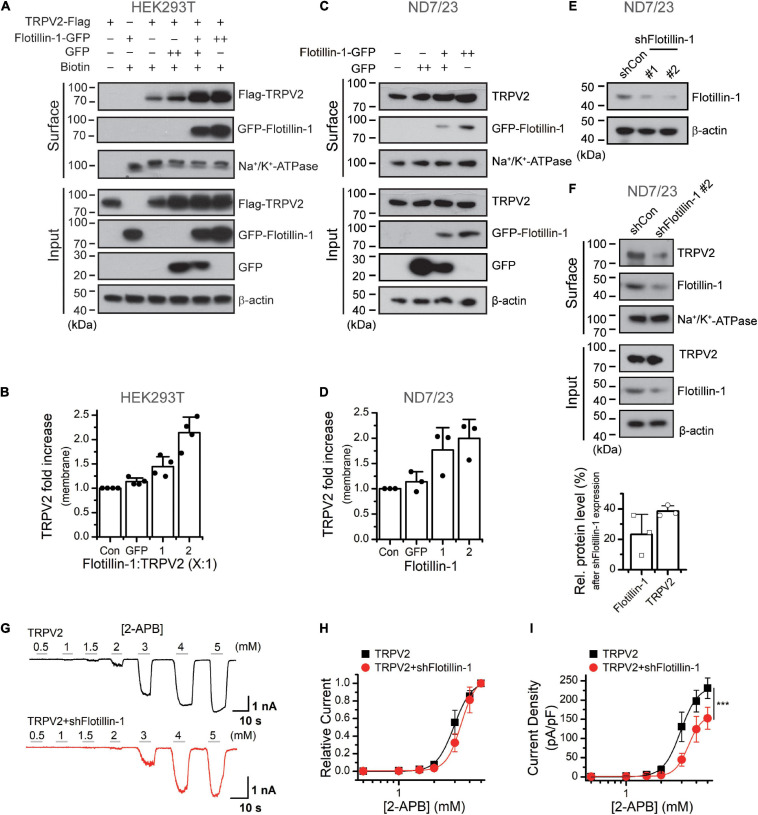
Flotillin-1 enhances the surface expression levels of TRPV2. **(A)** Increased surface expression of TRPV2 by flotillin-1. HEK 293T cells were transiently transfected with TRPV2 with GFP or increased flotillin-1 as indicated. Surface levels of TRPV2 were measured by IB after plasma membrane proteins were biotinylated and purified with NeutrAvidin agarose beads. β-actin and Na^+^/K^+^ ATPase were used as controls for cytoplasmic and membrane proteins, respectively. **(B)** Quantitative analysis of the fold increase of TRPV2 on the plasma membrane by co-expression with flotillin-1 in HEK 293T cells (*n* = 4; means ± SD). **(C,D)** ND7/23 cells were transiently transfected with increasing amounts of flotillin-1-GFP cDNA. Similarly, surface levels of TRPV2 were measured by IB after plasma membrane proteins were biotinylated and purified with NeutrAvidin agarose beads. Blots are representatives of three independent experiments. Error bars represent SD. **(E)** Immunoblot analysis (with anti-flotillin-1) of ND7/23 cells transfected for 36 h with plasmids encoding flotillin-1-targeting shRNA (shFlotillin-1 #1, and shFlotillin-1 #2) or control shRNA (shCon) to test efficiency of shRNA. **(F)** Effect of knocking down flotillin-1 on the surface expression of TRPV2 in ND7/23 cells. The cell surface biotinylation assay was used to measure the surface levels of TRPV2 in ND7/23 cells that were stably transfected with shCon or shFlotillin-1 #2. Below showing the quantitative analysis of the relative protein levels of TRPV2 and flotillin-1 caused by shFlotillin-1 #2. Data are representative of three independent experiments. **(G)** Whole-cell recordings in HEK 293T cells expressed TRPV2 alone (*upper*), and TRPV2 + shFlotillin-1 (*lower*), showing that 2-APB dose-dependently activated TRPV2 channels. Holding potential was –60 mV. **(H)** Dose–response curves for 2-APB-evoked currents. Solid lines indicate fits with the Hill equation, which yielded EC_50_ = 2.95 ± 0.06 mM, *n*_*H*_ = 5.84 ± 0.72 for TRPV2 (*n* = 8); and EC_50_ = 3.39 ± 0.02 mM, *n*_*H*_ = 6.87 ± 0.17 for TRPV2 + shFlotillin-1 (*n* = 8). **(I)** Summary plot of current density. The current densities evoked by 2-APB were determined by normalizing the membrane peak current by membrane capacitance. (*n* = 8). ***P < 0.001.

The increase in whole-cell current density suggests an enhanced surface expression levels of the ion channel. We then explored the influence of flotillin-1 on TRPV2 surface expression level. Flotillin-1-GFP and TRPV2-Flag were co-expressed in HEK 293T cells. The amounts of surface-expressed and total Flag-TRPV2 proteins were determined by surface biotinylation and western blotting, respectively. As revealed in Figure 4A, along with the amount of transfected flotillin-1 increased, the expression level of TRPV2 on the plasma membrane was proportionally upregulated. In this condition, however, the total amount of TRPV2 was unaffected. Since flotillin-1 is expressed on the inner side of the membrane, it cannot be directly biotinylated in the case of no TRPV2 expression (Figure 4A). Being co-expressed with TRPV2, flotillin-1 turned to be biotinylated as expected by their physical interaction (Figure 4A). With the sufficiently increased expression of flotillin-1, TRPV2 surface expression showed a nearly twofold increase compared with the control group (Figure 4B). In addition, we explored the effect of overexpressed flotinllin-1 on TRPV2 protein expression in ND7/23 cells, a hybridization line of mouse neuroblastoma and rat DRG neuron (Wood et al., 1990), that express endogenous TRPV2. As illustrated in Figures 4C,D, transfection of flotinllin-1 in ND7/23 cells proportionally increased the surface expression, but not the total amount of TRPV2 channel. These findings are consistent with the increased TRPV2 current density following flotillin-1 overexpression (Figure 2). Next, we evaluated the regulatory effect of endogenous flotillin-1 on TRPV2 expression using shRNA-mediated knockdown. As shown in Figure 4E, shFlotillin-1 #2 could remarkably inhibit the expression of endogenous flotillin-1, we used it for the following experiments. Then the surface biotinylation experiment was performed in ND7/23 cells to detect the shRNA impact on the total and surface expression of TRPV2. Quantitative analysis showing that ∼80% reduction in flotillin-1 protein levels by transfection with shFlotillin-1 #2. As expected, the knockdown of endogenous flotillin-1 indeed reduced the surface expression of TRPV2 (Figure 4F). We further examined the effects of flotillin-1 knockdown on the electrophysiological activity of TRPV2. As illustrated in Figures 4G,H, we compared TRPV2 function by activating the channel with increasing concentrations of 2-APB at the holding potential of −60 mV. Fitting the dose-response curves with the Hill equation yielded similar EC50 values and Hill coefficients (*n*_*H*_) for TRPV2 co-expressed with shFlotillin-1 #2 or not (EC50 = 2.95 ± 0.06 mM, *n*_*H*_ = 5.84 ± 0.72 for TRPV2, *n* = 8; and EC50 = 3.39 ± 0.02 mM, *n*_*H*_ = 6.87 ± 0.17 for TRPV2 + shFlotillin-1 #2, *n* = 8). The current density, however, showed a significant difference, e.g., 230.9 ± 26.8 pA/pF (*n* = 8) for TRPV2 and 152.6 ± 28.6 pA/pF (*n* = 8) for TRPV2 + shFlotillin-1 #2 evoked by 5 mM 2-APB, respectively (Figure 4I). Taken together, our results suggest that flotillin-1 exerts a role in regulating the surface expression levels of TRPV2, thereby its whole-cell current density.

### Flotillin-1 Sustains the Surface Stability of TRPV2

**FIGURE 5:**
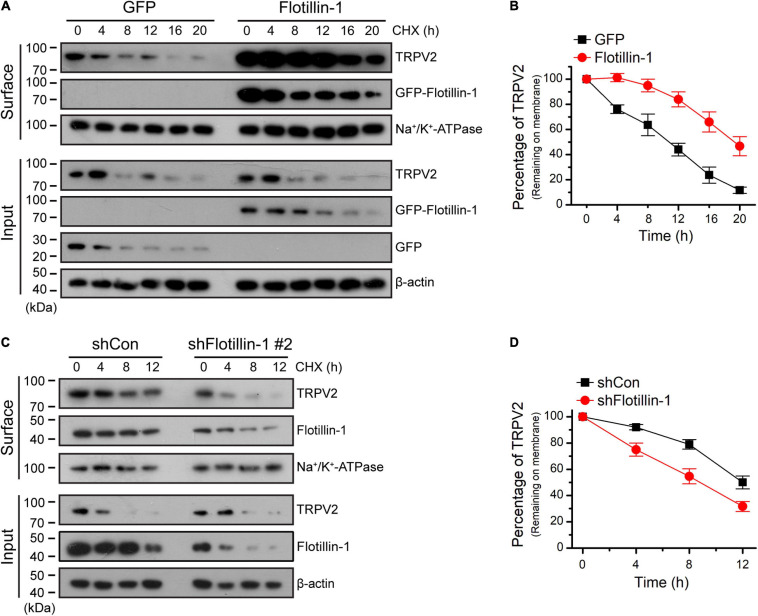
Flotillin-1 sustains the surface stability of TRPV2. **(A)** Immunoblotting analysis of TRPV2 in ND7/23 cells time-dependently treated with the protein synthesis inhibitor cycloheximide (CHX, 100 μg/ml) after being transiently transfected with GFP-tagged flotillin-1 or GFP. After plasma membrane proteins were biotinylated and purified with streptavidin-agarose, the surface levels of TRPV2 were analyzed by IB. **(B)** Quantitation of the surface levels of TRPV2 after treatment using CHX. Quantitation was performed by digitizing the immunoblots. **(C)** CHX-chase experiment assessing the surface stability of TRPV2 in ND7/23 cells that expressed shCon or shFlotillin-1 #2. Cells were treated with 100 μg/ml of CHX for indicated times. **(D)** Quantification of the surface levels of TRPV2, showing a marked accelerate in endogenous TRPV2 degradation kinetics by knocking down flotillin-1 when compared to control cells. Data are representative of three independent experiments.

To understand the effect of filotillin-1 on TRPV2 surface expression, we examined the steady-state protein level and the turnover rate. ND7/23 cells were transfected with flotillin-1-GFP or the GFP control vector. The stability of TRPV2 was measured by immunoblotting after treatment with the protein synthesis inhibitor cycloheximide (CHX, 100 μg/mL). In the absence of flotillin-1, TRPV2 is relatively unstable, showing a degradation kinetics that reaches ≥80% elimination upon 20 h translational arrest. On the contrary, in the presence of flotillin-1 the channel exhibited significantly slower elimination kinetics and reduced degradation rate, as only about half of TRPV2 faded away after 20 h (Figures 5A,B). We next sought to examine the degradation rate of surface-expressed TRPV2 following shRNA-mediated flotillin-1 knockdown. As expected, with flotillin-1 knockdown, the membrane TRPV2 exhibited a faster degradation rate (Figures 5C,D). Thus, flotillin-1 plays a role in sustaining TRPV2 protein stability and facilitates its surface expression.

### Mapping of the Interaction Domains Between TRPV2 and Flotillin-1

**FIGURE 6:**
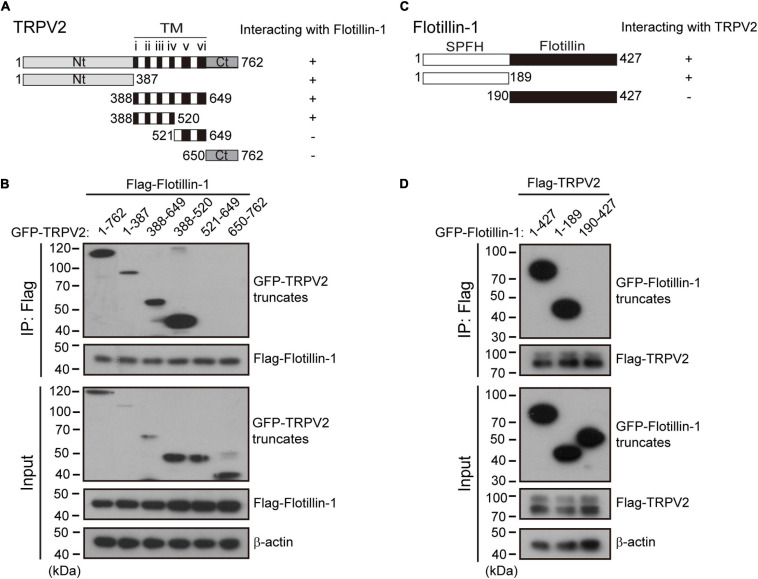
Identification of interaction domains for the association between TRPV2 and flotillin-1. **(A)** A schematic representation of full-length TRPV2, its mutants that were studied, and their abilities to interact with flotillin-1. The number denotes the position of the amino acid residues. **(B)** N-terminal region and transmembrane domains 1–4 of TRPV2 are responsible for the interaction between TRPV2 and flotillin-1. HEK 293T cells were transiently transfected with different truncation mutants tagged with GFP and flotillin-1-Flag as indicated. Cell lysates were immunoprecipitated with anti-Flag agarose beads and analyzed by IB using anti-GFP and anti-Flag, respectively. Whole-cell lysates were also used for IB with anti-GFP and anti-Flag for input. **(C)** Schematic diagram of composition of mutant flotillin-1 including SPFH domain (aa 1–189), and flotillin domain (aa 190–427), and as well as their abilities to interact with TRPV2. **(D)** HEK 293T cells were transfected with TRPV2-Flag and different mutant flotillin-1 fused with GFP as indicated. Cell lysates were subjected to IP by anti-Flag, followed by IB for anti-GFP and anti-Flag, respectively. Note, molecular weight standards (in KD) are shown on the left. TM, transmembrane. Data are representative of three independent experiments.

Next, we determined which molecular regions of TRPV2 and flotillin-1 proteins were responsible for their interaction. Structurally, TRPV2 has six transmembrane (TM) domains flanked by intracellular N- and C-terminal domains, and a short pore-forming loop between the fifth (TM5) and sixth (TM6) transmembrane. In order to define the regions interacting with flotillin-1, we constructed various GFP-tagged deletions of TRPV2 as illustrate in Figure 6A. They were then individually co-expressed with FLAG-tagged-flotillin-1 in HEK 293T cells for co-IP evaluation. Flotillin-1 was strongly precipitated with TRPV2 N-terminus (aa 1–387) and transmembrane domains 1–4 (aa 388–520), respectively. In contrast, neither the pore region (aa 521–649) nor C-terminus (aa 650–762) showed interaction with flotillin-1, indicating N-termius and TM 1–4 of TRPV2 are required for the protein interaction (Figure 6B). Similarly, we further mapped the molecular domains of flotillin-1 that mediated its interaction with TRPV2. As shown in Figure 6C, two truncations of flotillin-1 were constructed, including SPFH domain (aa: 1–189), and Flotillin domain (aa: 190–427). Figure 6D shows that TRPV2-Flag could be precipitated by the SPFH domain, but not the Flotillin domain. These results thus indicate that the SPFH domain of flotillin-1 and the N-terminus and TM 1–4 of TRPV2 are required for their interaction.

## Discussion

The regulatory interactions between TRP channel and signaling molecules, and the formation of these protein complexes have a significant effect on their functions (Barnhill et al., 2004; Stokes et al., 2004; Zhang et al., 2008; Kim et al., 2011). Thus, the identification and characterization of TRPV2-interacting proteins are pivotal for a better understanding of the physiology of this thermo TRP channel. Here, we demonstrate that the scaffold protein flotillin-1 directly complexes with TRPV2 in both heterologous cell expression system and native DRG sensory neurons. The N-terminus (aa 1–387), the region spanning the first to fourth transmembrane domains (aa 388–520) of TRPV2 and the SPFH domains of flotillin-1 (aa 1–189) mediate their interaction. Functionally, flotillin-1 facilitates the cell surface expression levels of TRPV2, thereby enhancing the whole-cell current density. These findings broaden our understanding of the regulatory mechanisms of TRPV2 signaling complex.

Flotillin-1 is preferentially associated with flotillin-2 in hetero-oligomeric complexes, forms membrane microdomains that serve as scaffolds facilitating the assembly of multiprotein complexes at the membrane-cytosol interface (Zhao et al., 2011; Kwiatkowska et al., 2020). It was observed that flotillin-1 is associated with tyrosine-protein kinase Lyn and enhances its activity (Kato et al., 2006). The interaction between flotillin-1 and neuroglobin (Ngb) implied that flotillin-1 might play a crucial role in regulating neuronal death and proliferation (Wakasugi et al., 2004). As mentioned above, previous studies have shown that flotillins modulate several ion channels activity via the interplay between each of them. For instance, the reduction of potassium channel Kv2.1 current amplitude by flotillin-1 was suggested to be mainly due to the Kv2.1 clustering on the plasma membrane (Liu et al., 2016). In contrast, Kessler et al. (2019) reported that both protein expression and channel activity of Nav1.5 channel were significantly decreased in *flotillin-1* or *flotillin-1/2* deficient mice, thereby reducing cardiac excitation. Here we observed that the presence of flotillin-1 enhanced the stability of TRPV2 channels on the plasma membrane, thereby up-regulating their responsive current density.

Flotillin-1 and flotillin-2 are two ubiquitous, highly conserved homologous proteins that assemble to form heterotetramers on the inner surface of cell membranes in cholesterol- and sphingolipid-enriched domains (Gauthier-Rouvière et al., 2020). The heterotetramers not only act as a skeleton to provide a platform for protein–protein interactions, but also are involved in signal transduction, nerve regeneration, endocytosis, and lymphocyte activation (Solis et al., 2010). Flotillins are composed of two domains: the N-terminal SPFH domain associated with the inner leaflet of cell membranes and the C-terminal domain is required for oligomerization (Morrow et al., 2002; Neumann-Giesen et al., 2004). The fact that TRPV2 shows no interaction with flotillin-2 but specifically interacts with the SPFH domain of flotillin-1, suggests that these two proteins might have distinct features in their SPFH domain or their C-terminal associations.

Studies have demonstrated that ankyrin repeat sequence motifs are common protein–protein recognition domains and, they are clearly important for the modulation of TRP channels. Moreover, the membrane proximal or pre-S1 domain of TRPV2 has been suggested to mediate protein–protein and lipid–protein interactions (Donate-Macian et al., 2019). Neeper et al. (2007) reported that deletion of 83 or more N-terminal residues greatly decreased TRPV2 expression levels. In addition to these observations, recently, it has been revealed that two structural motifs in transmembrane segments 2 and 4 of mTRPA1 regulates its interaction with cholesterol, which appears necessary for normal agonist sensitivity and plasma membrane localization (Startek et al., 2019). Consistent with these observations, we have shown here that the N-terminal and TM1–4 of TRPV2 are essential for the interactions between flotillin-1 and TRPV2. This observation parallels our finding that flotillin-1 helps to stabilize TRPV2 membrane expression.

It has been reported that flotillin-mediated endocytosis and ALIX–syntenin-1–mediated exocytosis protect the cell membrane from damage caused by necroptosis (Fan et al., 2019). Given the role of flotillin-1 in protein clustering and endocytosis, it is also plausible that flotillin-1 plays a role in maintaining the normal recycling of TRPV2 to the plasma membrane. This process would be important for refreshing the functional pool of TRPV2 on cell surface. We can not exclude the possibility that flotillin-1 may negatively regulate the intracellular degradation of TRPV2. This would also explain that an overexpression of flotillin-1 enhances TRPV2 membrane expression, and vice versa.

Since TRPV2 channel has been implicated in the processing of high intensity thermal pain, mechano-sensation, regulation of cardiovascular function, as well as many aspects of pathophysiology in different ways (Peralvarez-Marin et al., 2013; Shibasaki, 2016), our findings support that flotillin-1 is a key molecular element in TRPV2 signaling complex and exerts a regulatory effect on its cellular response. Targeting flotillin-1 would offer an updated intervention strategy to manage TRPV2-mediated physio- and pathology responses.

## Conclusion

We have characterized the role of flotillin-1 in mediating the membrane expression and cellular responses of TRPV2. This functional crosstalk between TRPV2 and lipid raft components may influence the cellular morphology and play critical roles in nociception and pain.

## Data Availability Statement

The original contributions generated for this study are included in the article/supplementary material, further inquiries can be directed to the corresponding author.

## Ethics Statement

All mice were housed in the specific pathogen-free animal facility at Wuhan University and all animal experiments were in accordance with protocols approved by the Institutional Animal Care and Use Committee of Wuhan University (No. WDSKY0201804).

## Author Contributions

JY designed and supervised the study. JH, YG, QH, YW, XM, PW, YZ, CX, and JY carried out the experiments and analyzed the data. DL provided technical support and suggestions. JH and JY wrote the manuscript with inputs from all other authors. All authors discussed the results and commented on the manuscript.

## Conflict of Interest

The authors declare that the research was conducted in the absence of any commercial or financial relationships that could be construed as a potential conflict of interest.
